# Factors associated with the consumption of ultra-processed food by Brazilian adolescents: National Survey of School Health, 2015

**DOI:** 10.1590/1984-0462/2022/40/2020362

**Published:** 2021-10-04

**Authors:** Janiquelli Barbosa Silva, Bianca Caroline Elias, Sarah Warkentin, Laís Amaral Mais, Tulio Konstantyner

**Affiliations:** a Universidade Federal de São Paulo, São Paulo, SP, Brazil.

**Keywords:** Adolescent, Food consumption, Industrialized foods, Health surveys, Adolescente, Consumo alimentar, Alimentos industrializados, Inquéritos epidemiológicos

## Abstract

**Objective:**

To identify the prevalence and factors associated with the consumption of ultra-processed foods by Brazilian adolescents.

**Methods:**

The sample was representative of adolescents and participants in the cross-sectional population-based study National Survey of School Health, 2015 edition (PeNSE-2015). A self-administered questionnaire was used for data collection. The variable weekly consumption of ultra-processed foods was considered, and consumption more than seven times a week was considered excessive. Descriptive and inferential analyses of demographic, socioeconomic, behavioral and environmental characteristics potentially associated with the outcome were performed. Poisson's multiple regression model was adjusted to control for confounding factors.

**Results:**

The prevalence of excessive consumption of ultra-processed foods among 16,324 adolescents in Brazil was 75.4%. Nine factors independently associated with this outcome were identified: age under 15 years (RR 1.08; p<0.001), daily sitting time greater than four hours (RR 1.13; p<0.001), eating while watching TV or studying more than four days a week (RR 1.09; p<0.001), daily TV time greater than three hours (RR 1.08; p<0.001), breakfast frequency less than four days a week (RR 1,03; p=0.001), having a cell phone (RR 1.12; p<0.001), absent maternal education (RR 0.88; p<0.001), being enrolled in a private school (RR 1.05; p=0.002) located in the urban area (RR 1.13; p=0.002).

**Conclusions:**

The results express the multifactorial characteristic of excessive consumption of ultra-processed foods and suggest the need for the development and implementation of health policies to guide the consumption of these foods and the importance of adopting healthy behaviors for this population group in both school and home environments.

## RESUMO

**Objetivo:**

Identificar a prevalência e os fatores associados ao consumo de alimentos ultraprocessados em adolescentes brasileiros.

**Métodos:**

Estudo transversal de base populacional com adolescentes participantes da Pesquisa Nacional de Saúde do Escolar, 2015 (PeNSE-2015). Um questionário autoaplicável foi utilizado para a coleta de dados, e construiu-se uma variável representativa do consumo de alimentos ultraprocessados, sendo considerado excessivo o consumo superior a sete vezes na semana. Realizou-se análise descritiva e inferencial das características demográficas, socioeconômicas, comportamentais e ambientais potencialmente associadas ao consumo de alimentos ultraprocessados. Um modelo de regressão de Poisson múltiplo foi ajustado para controle do confundimento.

**Resultados:**

A prevalência do consumo excessivo de alimentos ultraprocessados nos 16.324 adolescentes estudados no Brasil foi de 75,4%. Identificaram-se nove fatores associados de forma independente a esse desfecho: idade inferior a 15 anos (RR 1,08; p<0,001), tempo diário sentado superior a quatro horas (RR 1,13; p<0,001), comer assistindo à TV ou estudando por mais de quatro dias na semana (RR 1,09; p<0,001), tempo diário de uso de TV superior a três horas (RR 1,08; p<0,001), frequência de desjejum inferior a quatro dias semanais (RR 1,03; p=0,001), possuir telefone celular (RR 1,12; p<0,001), escolaridade materna ausente (RR 0,88; p<0,001), estar matriculado em escola privada (RR 1,05; p=0,002) e localizada em zona urbana (RR 1,13; p=0,002).

**Conclusões:**

Os resultados expressam a característica multifatorial do consumo de alimentos ultraprocessados e sugerem a necessidade de elaboração e execução de políticas de saúde para orientar sobre os prejuízos do consumo desses alimentos e a importância da adoção de comportamentos saudáveis para esse grupo populacional no ambiente escolar e familiar.

## INTRODUCTION

Overweight in adolescence is associated with several metabolic and cardiovascular changes and represents a public health problem with high prevalence in Brazil and worldwide. Between 1975 and 2016, a global increase in the number of children and adolescents (5–19 years) was estimated in approximately 113 million cases.^1^ ^,^ ^2^ Brazilian population-based surveys indicate prevalence of overweight ranging from 15.3 and 20.5% in this age group.^1^ Caloric intake from foods of low nutritional quality and physical inactivity contribute significantly to these rates.^1^ ^–^ ^3^

Ultra-processed foods (UPF) are among these foods, and, according to the NOVA classification, they are industrial formulations rich in sugars, fats and sodium and low in micronutrients, bioactive compounds and fibers. In addition, they have attractive characteristics such as high palatability, sophisticated packaging, good marketing and ease of access, which encourage excessive consumption and substitution of traditional food kinds.^5^ ^,^ ^6^

The inclusion of these products in the Brazilian diet has been associated with an increasing trend.^7^ Soft drinks and other types of sugar-sweetened beverage, sausages, stuffed cookies, snacks, treats, instant noodles, fried and roasted pizzas and snacks are among the most consumed UPF by adolescents.^8^ ^,^ ^9^ Contrarily, traditional foods such as beans, milk, fruits and vegetables are less and less present in the diet of young people.^8^ ^,^ ^10^ The Family Budget Survey (2017–2018) identified that about 26.7% of the total caloric intake by adolescents in Brazil came from UPF, the highest percentage when compared to the group of adults and the elderly.^9^

Inadequate eating practices at this stage of life can lead to nutritional deficiencies and affect the growth process inherent in puberty. In addition, they contribute to the development of health problems and long-term worsening of quality of life.^1^ ^,^ ^3^

Given the multifactorial characteristic food consumption, our hypothesis is that some factors associated with a higher frequency of consumption of UPF may be modifiable. Consequently, their identification would help to develop strategies for food and nutrition education and prevention of overweight.

This study, therefore, aimed to estimate the prevalence and identify factors associated with the consumption of UPF in a representative sample of Brazilian adolescents.

## METHOD

This study used secondary data from the National School Health Survey, 2015 edition (PeNSE-2015), a population-based survey with cross-sectional design carried out by the Ministry of Health of Brazil (MS) in partnership with the Brazilian Institute of Geography and Statistics (IBGE). The database is publicly accessible and available electronically on the IBGE website.^11^

The target population of the study were schoolchildren enrolled in the 6th to the 9th grades of elementary school and in the 1st to the 3rd grades of high school, from morning, afternoon and evening shifts, in public and private schools of urban and rural areas of Brazil. The selection allowed to estimate the geographic and population scope, representing Brazil and its five geographic macro-regions. Based on the 2013 School Census, schools with at least 15 students enrolled in the series of interest were eligible.^11^ All 16,556 individuals from sample 2 of PeNSE-2015 were studied. This sample represents our target population and is composed of students who answered the questionnaire and who had their weight and height measured during data collection.

All the information was collected in a self-administered questionnaire structured in thematic modules addressing socioeconomic, behavioral, dietary and health aspects. The questions were of multiple choice and the student had the choice of not answering any of the questions or the whole questionnaire. Food consumption was measured using the frequency of consumption in the week prior to the survey date, varying from zero to seven days for each food group.^11^

We investigated the existence of a pattern of food consumption as an effect of the joint perception of consumption and interaction of behaviors related to food, generally not perceptible through instruments of food data collection.^12^

To define the patterns of food consumption, an exploratory factor analysis (EFA) was applied with the Promax orthogonal rotation method, which considers a correlation between the factors studied. So, food groups were created according to the frequency of consumption. As indicators of model quality, the following were calculated: the Cronbach's alpha coefficient, to analyze the internal consistency of variables (0.683), the Kaiser-Meyer-Olkin measures, to assess factorial adequacy (0.772), and the Bartlett test, to analyze the correlation between variables (p<0.001). The results indicated sufficient and adequate correlation conditions to proceed with the analysis. Factor loads greater than 0.30 were considered significant in the correlation matrix.^13^

The application of EFA based on the eight food categories included in the questions about frequency of food intake (beans, fruits, vegetables, soft drinks, sweets, fried snacks, salted processed foods and fast food) resulted in the identification of a single food pattern. Based on the NOVA classification, which categorizes foods by the extent and purpose of industrial processing,^5^ the five food groups that made up the pattern identified were all UPF (fried snacks, salted processed foods, soft drinks, sweets and fast food).

These foods showed the greatest correlations between real consumption and the joint perception of consumption. The factorial loads obtained were: fried snacks (0.574), salted processed foods (0.552), soft drinks (0.535), sweets (0.523) and fast food (0.484). The frequency of consumption of each of the five food groups was weighted by the value of their factor load to obtain the representative variable of UPF consumption in the sample.

The consumption of UPF varied from zero to 35 times a week, that is, the possibility of consumption was from none to five food groups per day, seven days a week. Excessive consumption of UPF was attributed to frequency more than seven times a week, based on the distribution of consumption in the studied sample (25th percentile). Since the Food Guide for the Brazilian Population by the Ministry of Health recommends avoiding the consumption of UPF,^6^ we considered acceptable to consume weekly less than or up to seven foods belonging to one of the five groups of UPF in our analysis.

A variable was specifically created to represent the consumption of fresh or minimally processed foods (MPF) based on the average weekly consumption of beans, fruits and vegetables. Consumption less than five times a week was considered inadequate. In addition, we studied the weekly frequency of consumption of school meals, breakfast, main meals (lunch and dinner) in the presence of parents/guardians and meals in front of the TV or while studying.

In addition, the level of physical activity measured through the average weekly time accumulated in minutes was assessed based on the time spent commuting to and from school on foot or by bicycle, in activities in physical education classes and outside school, including sports, dance, gymnastics, weight training and fights.^11^ Subjects with an accumulated average weekly time of more than 300 minutes and “insufficiently active/inactive” were those reporting less than 300 minutes.

Finally, we used the body mass index (BMI) and the classification of the nutritional status available in the PeNSE-2015 database, according to the Z score of BMI-for-age based on the criteria of the World Organization of Health (WHO).^11^ “Nutritional deficit” was defined as values less than −2 standard deviations (SD), “normal weight” values between −2 and 1 SD, “overweight” values greater than 1 to 2 SD and “obesity” values greater than 2 SD.^11^ The cutoff points proposed for the other variables were defined by the distribution characteristic or by the form of categorization in the questionnaire.

Data were analyzed in the software Stata 14.0 (StataCorp LLC, College Station, USA). We considered the weight and sample expansion according to the selection process and population representativeness proposed by PeNSE-2015.^11^

Pearson's chi-square test was used to compare the prevalence of excessive consumption of UPF between regions. Unadjusted and adjusted Poisson regression analyses were used to independently identify the factors associated with excessive consumption of UPF. In order to select the variables eligible to compose the multiple model, the value of p≤0.20 was considered as inclusion criterion. The variable entry technique used was the Stepwise Forward, and the value of p<0.05 was used to define a statistically significant association.

PeNSE-2015 was approved by the National Research Ethics Commission of the Ministry of Health under opinion 1,006,467. Students who voluntarily agreed to participate and signed the free and informed consent form were included in the research.^11^ This study was approved by the Research Ethics Committee of Universidade Federal de São Paulo (Opinion No. 2,608,318).

## RESULTS

All 16,556 students who made up sample 2 of PeNSE-2015 were evaluated. Only 232 were excluded from the multiple analysis due to missing data regarding the selected variables to compose the final model. Therefore, 16,324 participants were added in the multiple analysis, with a sample loss of 1.4%.

**Figure 1:**
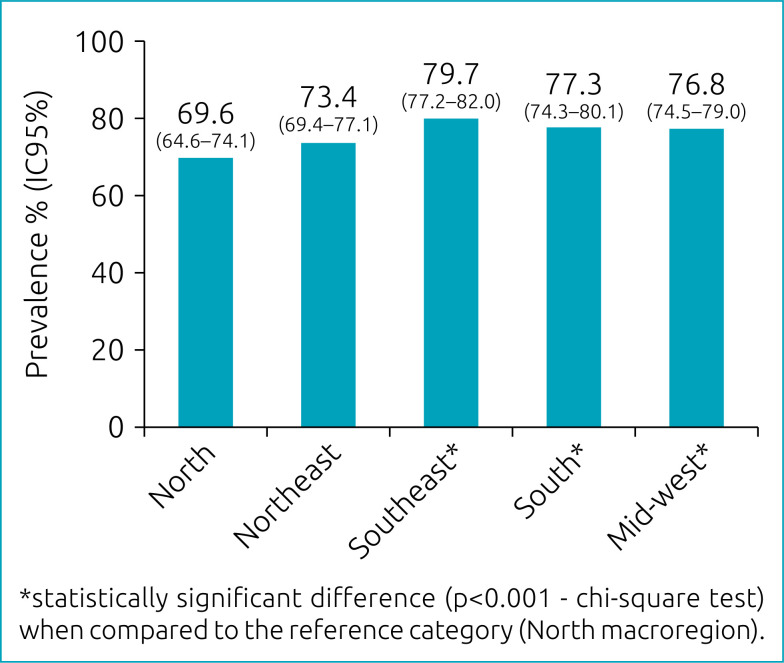
Prevalence of consumption of ultra-processed foods among adolescents participating in the National School Health Survey 2015 in the five Brazilian macro-regions.

The prevalence of excessive consumption of UPF estimated in Brazil was 75.4% (95%CI 73.3–77.3). This prevalence varied significantly between the five Brazilian macro-regions (p<0.001). The highest and lowest consumptions were estimated in the Southeast and North regions of Brazil, respectively (Figure 1).

Of the total participating adolescents, 18.9% lived in the North macro-region, 20.5% in the Northeast, 20.1% in the Southeast, 20.0% in the South, and 20.5% in the Midwest region. Regarding accumulated physical activity, 75.2% were classified as inactive/insufficiently active and 24.8% as active. As for nutritional status, 2.2% were underweight, 63.6% were eutrophic and 34.2% were overweight. Table 1 lists their other descriptive characteristics.

**Table 1:** Prevalence and 95% confidence interval of the clinical, behavioral and epidemiological characteristics of Brazilian adolescents, National Student Health Survey 2015.

			n	%	95%CI
Socioeconomic and demographic characteristics					
	Administrative dependency of the school	Private	16,556	25.6	(13.7–42.7)
		Public		74.4	(57.3–86.3)
	School location	Urban	16,556	95.3	(91.2–97.5)
		Rural		4.7	(2.5–8.8)
	Full-time school shift	No	16,513	95.4	(94.1–96.4)
		Yes		4.6	(3.7–5.9)
	Sex	Female	16,556	48.1	(46.9–49.4)
		Male		51.9	(50.7–53.2)
	Skin color	White	16,533	40.1	(35.0–45.5)
		Other*		59.9	(54.5–65.1)
	Age	≥15 years	16,556	48.4	(38.1–58.8)
		<15 years		51.6	(41.2–61.9)
	Having a cell phone	Yes	16,538	88.0	(85.2–90.4)
		No		12.0	(9.6–14.8)
	Living with mother	No	16,545	11.5	(10.1–13.2)
		Yes		88.5	(86.8–89.9)
	Living with both parents	No	16,537	41.6	(39.3–44.0)
		Yes		58.4	(56–60.7)
	Number of people in household	≤4	16,541	62.7	(59.5–65.9)
		≥5		37.3	(34.1–40.5)
	Mother's schooling	Did not study	16,522	4.3	(3.3–5.6)
		Studied**		95.7	(94.4–96.7)
Eating behavior					
	Attitude towards own's weight	None	16,390	36.2	(34.5–37.9)
		Some***		63.8	(62.1–65.5)
	Frequency of breakfast	≤4 days	16,544	35.3	(33.0–37.7)
		>4 days		64.7	(62.3–67.0)
	Frequency of eating meals with father, mother or guardian	≤4 days	16,531	29.3	(26.9–31.7)
		>4 days		70.7	(68.3–73.1)
	Frequency of eating meals while watching TV or studying	>4 days	16,541	43.8	(41.3–46.3)
		≤4 days		56.2	(53.7–58.7)
	Frequency of meals offered by the school	<3 days	16,551	76.6	(72.3–80.4)
		≥3 days		23.4	(19.6–27.7)
	Presence of snack bar at school	Yes	16,556	57.0	(47.6–66.0)
		No		43.0	(34.0–52.5)
	Frequency of consumption of MPF	≤4 days	16,480	70.2	(68.2–72.1)
		>4 days		29.8	(27.9–31.8)
Use of public health service					
	Care at BHU in the last 12 months	Yes	16,328	49.8	(47.2–52.5)
		No		50.2	(47.5–52.8)
Sedentary behaviors					
	Daily TV time	More than 3 hours	16,506	39.1	(36.3–42.0)
		Up to 3 hours		60.9	(58.0–63.7)
	Daily sitting time	More than 4 hours	16,472	38.8	(36.9–40.7)
		Up to 4 hours		61.2	(59.3–63.1)

95%CI: 95% confidence interval;

* black, yellow, brown and indigenous;

** elementary school, high school, higher education: complete or incomplete;

*** reported attempt to maintain, lose or gain weight; MPF: fresh and minimally processed foods; BHU: basic health unit.

Of the total of 22 factors tested, 4 had no statistically significant association with excessive consumption of UPF: living with the mother (PR 1.01; p=0.491), MPF consumption (PR 1.00; p=0.782), being assisted in a basic health unit (UBS) in the last 12 months (PR 1.01; p=0.202), and accumulated physical activity (PR 1.00; p=0.975). Table 2 shows the crude analysis of the other 18 factors associated with this outcome.

**Table 2:** Crude Poisson regression with prevalence ratio and 95% confidence interval of factors associated with the consumption of ultra-processed foods by Brazilian adolescents, 2015 National School Health Survey.

		Categories	Excessive consumption of UPF	PR (95%CI)	p-value
Socioeconomic and demographic characteristics					
	Administrative dependency of the school	Private	78.8	1.06 (1.01–1.11)	0.011
		Public	74.2		
	School location	Urban	76.2	1.28 (1.18–1.40)	0.000
		Rural	58.9		
	Full-time school shift	No	75.7	1.08 (1.02–1.15)	0.008
		Yes	69.4		
	Sex	Female	75.8	1.01 (1.00–1.03)	0.121
		Male	75.0		
	Skin color	White	77.2	1.04 (1.01–1.08)	0.022
		Other*	74.2		
	Age	≥15 years	78.2	1.08 (1.05–1.11)	0.000
		<15 years	72.7		
	Having a cell phone Living with mother	Yes	76.9	1.19 (1.15–1.24)	0.000
		No	64.5		
		Yes	75.0		
	Living with both parents	No	76.4	1.03 (1.00–1.05)	0.033
		Yes	74.7		
	Number of people in household	≤4	76.0	1.02 (1.00–1.05)	0.039
		≥5	74.4		
	Mother's schooling	Did not study	62.7	0.83 (0.78–0.89)	0.000
		Studied**	76.0		
Eating behavior					
	Attitude towards own's weight	None	77.1	1.02 (1.00–1.05)	0.022
		Some***	74.4		
	Frequency of breakfast	≤4 days	78.5	1.07 (1.04–1.09)	0.000
		>4 days	73.7		
	Frequency of eating meals with father, mother or guardian	≤4 days	77.9	1.05 (1.02–1.08)	0.000
		>4 days	74.4		
	Frequency of eating meals while watching TV or studying	>4 days	80.6	1.13 (1.11–1.16)	0.000
		≤4 days	71.3		
	Frequency of meals offered by the school	<3 days	76.0	1.04 (1.01–1.08)	0.010
		≥3 days	73.4		
	Presence of snack bar at school	Yes	76.9	1.05 (1.02–1.09)	0.004
		No	73.4		
Sedentary behaviors					
	Frequency of consumption of MPF	More than 3 hours	81.2	1.14 (1.12–1.17)	0.000
		Up to 3 hours	71.7		
	Use of public health service	More than 4 hours	84.0	1.21 (1.18–1.24)	0.000
		Up to 4 hours	70.0		

95%CI: 95% confidence interval;

* black, yellow, brown and indigenous;

** elementary school, high school, higher education: complete or incomplete;

*** reported attempt to maintain, lose or gain weight; UPF: ultra-processed foods; PR: prevalence ratio. MPF: fresh and minimally processed foods.

**Figure 2:**
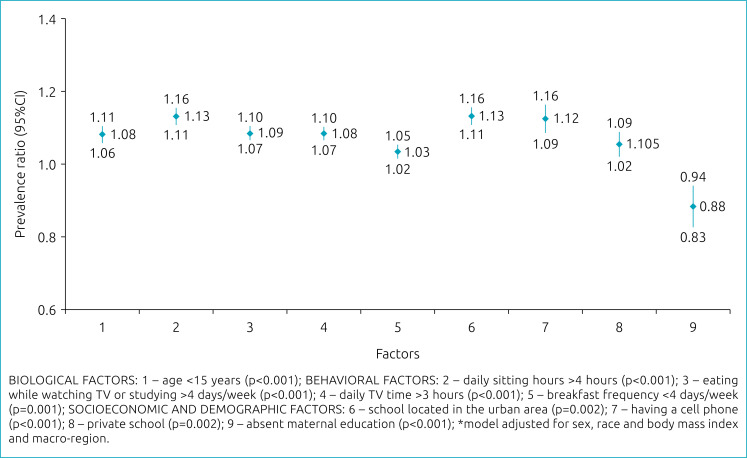
Multiple Poisson model of factors associated with the consumption of ultra-processed foods by Brazilian adolescents. National School Health Survey 2015 (n=16,324).

Figure 2 shows the factors that maintained a statistically significant association with excessive consumption of UPF after the assemblage of the final multiple regression model adjusted for macro-region, sex, skin color and BMI. These factors were grouped according to the dimension of determination:

1. biological factors: age;
2. behavioral factors: daily sitting hours, habit of eating meals in front of the TV or while studying, daily TV hours and frequency of breakfast;
3. socioeconomic and demographic factors: maternal education, school location, having a cell phone and administrative dependency of school.

## DISCUSSION

In our study, approximately three out of four adolescents presented excessive consumption of UPF. Nine factors were identified as independently associated with this outcome: eight risk factors (age less than 15 years, daily sitting time greater than four hours, eating while watching TV or studying more than four days a week, daily time watching TV higher than three hours, breakfast frequency less than four days a week, having a cell phone, studying at a private school located in the urban area) and a protection class (absent maternal schooling).

Behavioral, socioeconomic, cultural and environmental characteristics make up a complex network of determinants of UPF consumption by the Brazilian population.^14^ ^,^ ^15^ In addition to the unbalanced nutritional composition directly related to overweight and the development of chronic non-communicable diseases (NCDs), replacing traditional foods with UPF harms food culture, the environment and the individual's relationship with food and the society.^5^ ^,^ ^6^ ^,^ ^16^

In adolescence, changes in lifestyle, risky behaviors and the influence of social interactions are aspects that compromise the adoption of a healthy diet.^1^ ^,^ ^3^ The search for attractive, ready and easily accessible foods is seen as a convenient alternative for young people, leading to greater consumption of UPF at this stage of life.^3^ ^,^ ^16^ ^,^ ^17^

Due to the different experiences and situations in adolescence, the United Nations Children's Fund proposed to evaluate this period at two different times, before and after 15 years of age. In the initial phase, adolescents are looking for the construction of their identity, so analytical and reflective thinking is less comprehensive, which can lead them to adapt their behaviors as a form of acceptance and adaptation to the social environment.^18^ In this study, younger adolescents had a higher prevalence of excessive consumption of UPF. This finding can be explained by the fact that this group is potentially prone to being influenced by the social environment, has less critical capacity and less concern with food and the perception of body image when compared to older adolescents.^3^ ^,^ ^18^

Among the behavioral factors identified, the habit of sitting for more than four hours a day stands out, which included the use of screens and other electronic devices such as video games, cell phones and computers. The constant interaction of young people with the technological environment interferes decisively in the behaviors adopted by this population.^17^ ^,^ ^19^ The advertising of foods of low nutritional quality has reached different digital media of communication and entertainment frequently accessed by adolescents, which represents a scenario unfavorable for healthy food choices and, consequently, a possible facilitator of excessive consumption of UPF.^20^ ^,^ ^21^

In addition, the daily time spent watching TV of more than three hours was associated with excessive consumption of UPF, regardless of the sitting time. Watching TV for prolonged periods is a greater exposure to the negative effect of advertising of foods with low nutritional value in the formation of eating habits.^20^ ^–^ ^22^ According to a recent analysis of the programming of the four most watched open TV channels in Brazil, among the advertisements shown in the food and beverage category, the frequency of UPF exceeded the MPF by more than eight times, with special emphasis on sugary drinks and sweets.^23^

Although the factors previously mentioned may reflect sedentary leisure habits, our study showed that the practice of physical activity was not associated with the consumption of UPF independently. As pointed out in other studies with Brazilian adolescents,^4^ ^,^ ^21^ this means that sedentary habits are more determinant for the consumption of UPF among adolescents in PeNSE-2015 than not practicing physical activity as recommended (300 minutes).^11^

The habit of eating while watching TV or studying was also associated with excessive consumption of UPF. In addition to the distraction effect that these activities cause on satiety mechanisms, the presentation, practicality, palatability and ease attributed to the UPF stimulate the preference and excessive consumption.^6^ ^,^ ^15^ ^,^ ^16^ ^,^ ^20^ In fact, eating in front of the TV has been associated with a higher consumption of snacks, lower intake of micronutrients and higher intake of fats and sugars among adolescents.^22^ ^,^ ^24^

Breakfast is one of the most important daily meals, and the reduction in its frequency has been associated with a lower consumption of essential nutrients.^6^ ^,^ ^10^ In this study, a lower frequency of breakfast was associated with higher consumption of UPF. As in other developing countries in Latin America, in Brazil, among various types of UPF, the sale of ready meals, breakfast cereals and sugary drinks has increased alarmingly.^14^ ^–^ ^16^

Being enrolled in a private school was associated with excessive consumption of UPF and this can be explained by the frequent presence of snack bars and the lower supply of school meals in private educational institutions, contributing to a greater consumption of this type of food, such as industrialized snacks, sweets and soft drinks.^25^

This scenario may be the result of private schools not being obliged to adhere to the National School Feeding Program (PNAE), which has guidelines for promoting healthy eating in public basic education.^26^ Consequently, non-adherence to program recommendations by private institutions potentially reduces the incentive to food and nutrition education, contributing to the adoption of inappropriate eating practices. In addition, adolescents from private schools usually belong to families of higher socioeconomic status, which gives them greater possibility of access and consumption of UPF.

Although the consumption of UPF by adolescents from rural areas and small cities is of concern,^8^ ^,^ ^27^ those who were enrolled in schools located in the urban area showed a higher prevalence of excessive consumption of UPF in our analysis. Such association can be explained by the urban environment being a facilitator of the consumption of these foods, given the greater access, availability and variety of commercial establishments, products and brands in the market.^15^ ^,^ ^28^

Lower level of maternal education is associated with situations of risk to health and nutritional status in childhood, as it reflects less availability of resources for care and greater difficulty in accessing information.^29^ Although UPFs are associated with health problems, the results of our study reflect a characteristic of food consumption by adolescents, not the presence of diseases such as overweight. Thus, the absence of maternal education may be related to less purchasing power and restricts access to UPF. The higher level of education of mothers is generally associated with higher family income, which can contribute to a greater insertion of UPF in the meals’ routine.^14^ ^,^ ^15^

Finally, approximately nine out of ten adolescents in PeNSE-2015 reported having a cell phone at the time of the survey. This was also associated with excessive consumption of UPF. One possible explanation is that the use of this mobile device favors exposure to digital marketing of unhealthy foods through social networks and advertisements in various applications.^20^ ^,^ ^21^ In addition, young people who reported having this device probably integrate families with greater purchasing power, which is associated with greater access and consumption of UPF.^15^ ^,^ ^30^

It should be noted that the use of secondary data limited the analyses carried out to the information available in the PeNSE-2015 database. The amount consumed of available food groups, the consumption of other types of UPF and a specific income indicator were not evaluated. In this sense, the data collection carried out by means of a self-administered questionnaire could lead to a greater risk of errors in estimates. In addition, the associations do not allow the identification of causal relationships, since this study design does not allow the establishment of a temporal sequence of factors studied. Thus, the estimates and interpretation of associations found must be cautious.

On the other hand, PeNSE-2015 was a survey developed after a careful selection process of participating schools and with a representative sample of the Brazilian adolescent population. The EFA allowed to identify the sample's dietary pattern and enabled the creation of a variable that represented the real perception of UPF consumption among adolescents. In addition, the statistical analysis, carried out using a multiple model, allowed the control of confounding factors, which led to the identification of independent effects of the nine factors associated with excessive consumption of UPF, even though behavioral factors may express similar characteristics.

In this context, the results of this study indicate that excessive consumption of UPF is prevalent among Brazilian adolescents. The identification of nine associated factors suggests the need to implement actions to regulate UPF advertising and to promote healthy lifestyle habits with focus on reducing sedentary behaviors and on food and nutrition education in the school and family environment. Although these actions must be comprehensive, younger adolescents from private and urban schools should be prioritized.
